# School Refusal and Parental Illness: A Narrative Review

**DOI:** 10.1111/cch.70306

**Published:** 2026-06-19

**Authors:** Salomé Villetorte, Bruno Dauvier, Basilie Chevrier

**Affiliations:** ^1^ Aix Marseille Univ PSYCLE Aix‐en‐Provence France

**Keywords:** parental illness, risk factor, school refusal

## Abstract

**Introduction:**

School refusal (SR) affects many young people and has short‐ and long‐term consequences on their development. Early interventions improve SR prognosis. Among the risk factors studied, parental illness is often mentioned. This narrative review assesses the frequency and strength of the link between SR and parental illness and its specificities.

**Methods:**

PsycInfo, PsycArticles, Google Scholar and PubMed were queried, resulting in the inclusion of 18 studies.

**Results:**

This link exists, but the number of robust studies is limited. While often acknowledged, it is seldom considered the main topic and is more often found with parental mental illness than physical illness.

**Discussion:**

This association, observed in both quantitative and qualitative studies, seems to correspond to clinical reality. This review highlights the lack of precise data on parental illnesses in the SR literature and the limited hypotheses put forward to explain this link. This relationship requires greater attention in order to better understand and improve support for these children and adolescents and their families.

## Summary

- The association between school refusal and parental illness appears to be statistically significant and strong when studied.
- There is a lack of precise data on parental illnesses in the school refusal literature.
- Few and limited hypotheses on the explanatory factors of this link are proposed.
- The study of this link has theoretical and practical implications.
- These young people might be living in a parental environment that is not a source of support; therefore, this issue requires greater attention to better understand it and improve effective support for these children, adolescents and their families.

## 1 Introduction

School refusal (SR) affects around 4.8% of children and adolescents (Nair et al. 2013). It constitutes approximately 5% to 8% of child and adolescent psychiatry consultations, with variations across countries (Blandin et al. 2018; Suveg et al. 2005). SR currently represents a major issue worldwide, which affects the development of children and adolescents, as well as their families, with broader implications in legal and political domains (Golzálvez and Inglés 2019). Although commonly perceived as a new and increasing phenomenon, SR has existed since the establishment of mandatory education (Gallé‐Tessonneau and Dahéron 2022; Holzer and Halfon 2006). It was long assimilated to other school attendance problems (SAP) such as truancy, school withdrawal or school exclusion. Nonetheless, all of these SAP are characterised by diverse aetiologies and manifestations (Heyne et al. 2019).

The definition of SR proposed by Berg (1997), and subsequently modified by Heyne et al. (2019), is widely used and comprises four criteria: School refusal occurs when a young person (1) refuses to attend school while experiencing emotional distress, (2) does not try to hide absence from their parents, (3) does not display severe antisocial behaviour and (4) the parents make reasonable efforts to secure attendance at school (Heyne et al. 2019).

SR has multiple short‐ and long‐term consequences. In the short term, children and adolescents with SR are more likely to experience personal distress and to develop negative relationships with their family and peers (Ulaş and Seçer 2024). In the long term, they are at higher risk of psychiatric disorders, social adjustment difficulties, academic difficulties, reduced career opportunities and impaired emotional and intellectual development (Chockalingam et al. 2023; Kearney 2008). Nevertheless, early interventions improve SR prognosis (Richardson 2016).

To promote early interventions, numerous studies have investigated the risk factors associated with SR (Ulaş and Seçer 2024). SR is influenced by risk factors at cognitive, school and family levels (Gallé‐Tessonneau et al. 2017). Cognitive vulnerabilities include poor emotional regulation (Hughes et al. 2010) and negative thought patterns (Tekin et al. 2018). School risk factors contribute to SR through high academic expectations (Nursalim et al. 2018) and poor peer relationships (Havik et al. 2014). The family factors underlined by several authors (Chockalingam et al. 2023; Ingul et al. 2019) include disorganised attachment to the attachment figure (Brumariu and Kerns 2010), parental practices (Carless et al. 2015), intrafamilial communication deficit (Boussand et al. 2021) or parental illness (Bahali et al. 2011; Chockalingam et al. 2023). Descriptive analyses show that among a SR sample, between 14% and 18% of the children and adolescents had a parent with a physical illness and between 34% and 53% had a parent with a mental illness (McShane et al. 2001). Both parental physical and mental illness are acknowledged as risk factors to develop SR (Prabhuswamy 2018). Parental mental illness seems to be more often considered as a correlate of SR (Eroglu et al. 2024). The explanatory hypotheses underlying this link remain unclear, as illustrated by vague examples in the literature, such as children being affected by parental disease without further elaboration (Ulaş and Seçer 2024).

The objective of this narrative review is to explore the nature of the link between SR and parental illness in the literature and to investigate the specificities of this link such as the type of illness, the gender of the parent concerned by the illness, the potential explanatory factors and direction of this link.

## 2 Methods

Given the limited number of studies on this topic, a narrative review was conducted in order to explore the subject including a wide variety of studies (Sukhera 2022). This narrative study is part of the TOBIAS project (Trajectories of Educational Orientation, School Well‐Being and School Anxiety) which received ethics committee approval. As narrative reviews are inherently prone to bias such as selective inclusion and confirmation bias, we chose to broaden our perspectives by querying the following multiple electronic databases: PsycInfo, PsycArticles, Google Scholar and PubMed. No articles from the grey literature were found. Scientific articles written in French or English that mentioned SR and parental illness were detected using the search terms ‘school refusal’ or ‘refus scolaire anxieux’ AND ‘parent’. The period covered was 1997 (publication date of Berg's definition of SR) to November 2025. Records were identified through database searching on 6 November 2025 for PsycArticle and PsycInfo, 7 November 2025 for PubMed and 10 November 2025 for Google Scholar.

The selection process comprised the following steps: All titles and abstracts were screened for relevance; after excluding irrelevant articles, the full text of the remainder was read to control for the definition of SR and the mention of parental illness. The flexible nature of narrative review and the few number of studies on the subject allowed us to include quantitative studies, literature and systematic reviews, qualitative studies (case studies and reflexive articles). As SR is often presented as a school attendance problem and therefore mixed with absenteeism or truancy (Heyne et al. 2019), the selection took into account the definition of SR in the studies. While it is recognised that mothers of children with SR are in general more anxious and stressed than others (Eroglu et al. 2024), we focused in this review only on physical and mental illnesses above the pathological threshold. Moreover, we considered only the articles outside the COVID period as it is an extreme situational context, which may have exacerbated SR.

The quantitative studies reported coefficients of correlation and the significance of the results. When the articles included both clinical and control groups and reported *r* coefficients, Cohen's *d* was computed to assess the effect size of the difference between the two groups. In order to do so, the following mathematical formula *d* = 2*r*/√(1 − *r* ^2^) was applied to the studies using both a clinical and a control group. Cohen's *d* below 0.3 is considered small, moderate between 0.3 and 0.5 and strong above 0.5 (Cohen 1988).

## 3 Results

**FIGURE 1:**
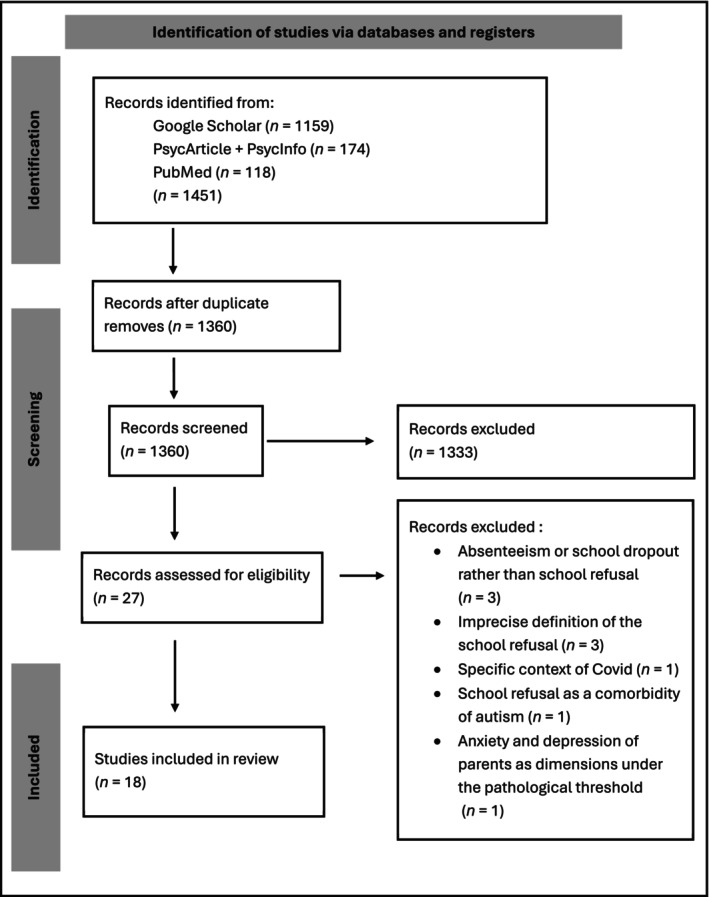
Flow diagram. Selection process of articles included in the systematic review.

The identification procedure led to 1451 articles screened. After removal of duplicates (91 articles) and of 1333 articles that did not meet the inclusion criteria, 27 reports were fully read and assessed for eligibility. Nine articles were excluded for the following reasons: The subject was absenteeism or school dropout rather than SR (*n* = 3; Allen et al. 2018; Kearney 2007; Zugaj et al. 2016); the definition of SR was imprecise (*n* = 4; Egger et al. 2003; Martin et al. 1999; Mullally and Connolly 2025); the study was specifically assessing the effect the Covid context of school refusal (*n* = 1; Matsubara et al. 2025); SR was a comorbidity of the autism spectrum (*n* = 1; Adams 2022); parental anxiety and depression were considered as dimensions and were under the pathological threshold according to the authors (*n* = 1; Eroglu et al. 2024). This resulted in a final total of 18 studies (see Figure 1 and Table 1).

**TABLE 1:** Nature of the article included, topic of parental illness, population surveyed, diagnostic of SR, type of parental illness, gender of the parent concerned by the illness, diagnosis of parental illness and quantitative results.

	Nature of the article	Topic of parental illness	Surveyed population (number of participants, age, gender)	Diagnostic of SR	Type of parental illness	Gender of the parent concerned by the illness	Diagnosis of parental illness	Quantitative results
Al Husni Al Keilani and Delvenne (2021)	Quantitative study	Acknowledged	Children and adolescents with SR (*n* = 71; 8–16) 70.42% male	Clinical team	Mental illness	Mothers 45.07% and fathers 28.17%	Hetero evaluation: medical records	/
Bahali et al. (2011)	Quantitative study	Principal	Parents of children with SR (*n* = 55) and without SR (*n* = 56) (< 5)	Clinical team	Mental and physical illness	Mothers (between 89 and 100%) and fathers 88%	Auto evaluation: Beck Depression Inventory (BDI; Beck et al. 1961), the State–Trait Anxiety Inventory (STAI; Spielberger et al. 1970), and the Symptom Checklist–90 revised (SCL90R; Derogatis 1977)	*d* = 1.10; *r* = 0.48; *p* < 0.001
Birraux (2014)	Qualitative study—Case study	Acknowledged	Adolescent with SR (*n* = 1; 16)	Clinical team	Mental illness (depression)	Mother	Hetero evaluation: psychologist	/
Carless et al. (2015)	Quantitative study	Secondary	Adolescents with SR and parents (*n* = 60; 12–17) 53% male	Clinical team	Mental illness (depression)	/	Auto evaluation: Beck Depression Inventory (BDI; Beck et al. 1961) and the State‐Trait Anxiety (STAI; Spielberger et al. 1970)	*r* = 0.44; *p* < 0.001
Chen et al. (2024)	Quantitative study	Secondary	Adolescents with (*n* = 30) and without SR (*n* = 76) and parents (12–18) 41.51% male	School attendance given by the school + absence of physical illness	Mental illness (anxiety and depression)	/	Auto evaluation: Depression Anxiety Stress Scales‐21 (DASS‐21; Lovibond and Lovibond 1995)	/
Chockalingam et al. (2023)	Quantitative study—Systematic review	Secondary	Children and adolescents with SR (4–19)	Studies mentioning Berg's definition	Mental illness	/	Auto evaluation: Symptom Checklist–90 revised (SCL90R; Derogatis 1977) and hetero evaluation: Structured Clinical Interview for the DSM‐IV Axis I Disorders (First et al. 1997)	/
Fremont (2003)	Qualitative study—Reflexive article	Acknowledged	/	/	Mental illness	/	/	/
Havik and Ingul (2021)	Qualitative study—Reflexive article	Acknowledged	/	/	Mental illness	/	/	/
Holzer and Halfon (2006)	Qualitative study—Reflexive article	Acknowledged	/	/	Not specified	/	/	/
Hughes et al. (2022)	Quantitative study	Secondary	Adolescents with SR and parents (*n* = 184; 10–17) 38% male	School Refusal Assessment Scale (SRAS‐C; Kearney 2002).	Mental illness (depression and anxiety)	/	Auto evaluation: The general depression scale of the Inventory of Depression and Anxiety Symptoms (IDAS‐GD; Watson et al. 2007)	/
Ingul et al. (2019)	Qualitative study—literature review	Acknowledged	Youth	/	Mental illness	/	/	/
Leduc et al. (2024)	Quantitative study—Systematic review	Acknowledged	Children and adolescents with SR (3–21)	Studies mentioning Berg's definition or that measure anxiety to identify SR	Mental and physical illness	/	/	/
Marin et al. (2019)	Quantitative study	Secondary	Anxious children and adolescents and parents (*n* = 343; 6–17) 49.6% male	Clinical team + the Anxiety Disorders Interview Schedule for Children—Parent Versions (ADIS‐IV: C; Silverman and Albano 1996)	Mental illness (depression)	/	Hetero evaluation: Anxiety Disorders Interview Schedule—Parent Versions (ADIS‐IV: P; Silverman and Albano 1996) Auto evaluation: Beck Anxiety Inventory (BAI; Beck et al. 1988) and Beck Depression Inventory (BDI; Beck et al. 1961)	*B* = 0.06; *p* = 0.01
Nuttall and Woods (2013)	Qualitative study—Case study	Acknowledged	Adolescents with SR (*n* = 2) and parents and professionals (13–14) girls	Clinical team	Not specified	/	/	/
Özcan et al. (2006)	Quantitative study	Principal	Children with (*n* = 25) and without SR (*n* = 25) and their parents (6–12) 56% male	Clinical team	Mental illness	Mothers 52% and fathers 36%	Hetero evaluation: Structured Clinical Interview for the DSM‐IV Axis I Disorders (First et al. 1997) Auto evaluation: Beck Anxiety Inventory (BAI; Beck et al. 1988); Beck Depression Inventory (BDI; Beck et al. 1961) and Liebowitz Social Anxiety Scale (LSAS; Liebowitz 1987)	*d* = 1.08; *r* = 0.46; *p* < 0.001
Prabhuswamy (2018)	Qualitative study—Reflexive article	Acknowledged	/	/	Mental and physical illness	/	/	/
Tekin and Aydın (2022)	Quantitative study—Systematic review	Acknowledged	Children and adolescents with SR (5–21)	/	Mental illness (depression and anxiety)	/	/	/
Ulaş and Seçer (2024)	Quantitative study—Systematic review	Acknowledged	Children and adolescents with SR	/	Mental illness (depression)	/	/	/

Among 1333 reports on SR and parental factors, only 18 acknowledged parental illness, indicating the low frequency with which it is addressed in the SR literature. This narrative review includes seven quantitative studies, five literature and systematic reviews and six qualitative studies (four reflexive articles and two case studies), which highlight the diversity of the data available on the subject. In general, the topic of parental illness is merely mentioned in passing (*n* = 11); occasionally, it is the secondary topic (*n* = 5) but very rarely the main topic (*n* = 2). The surveyed population is mostly clinical children and/or adolescents and their parents. The sex ratio of the children and adolescents is around 50%.

A link between parental mental illness and SR is more frequently observed (*n* = 13) than parental physical illness and SR (unspecified or both physical and mental illness, *n* = 5). The parental mental illnesses considered are depression and/or anxiety; no information on other mental illnesses is provided. Concerning parental physical illness, no information on diagnosis is given. The gender of the ill parent is rarely specified (*n* = 3). When gender is recorded, mothers appear to be slightly more likely to be ill than fathers. Diagnoses of parental illness are unspecified (*n* = 9), based on self‐evaluation with standardised psychometric scales (*n* = 5), other‐evaluation by the clinical team (*n* = 3) or both self‐ and other‐evaluations (*n* = 1).

Experimental studies showed significant differences in percentage of parental illness between the SR and the control groups (Bahali et al. 2011; *d* = 1.10; *r* = 0.48; *p* < 0.001; Özcan et al. 2006; *d* = 1.08; *r* = 0.46; *p* < 0.001). These effects are considered strong (Cohen 1988). Other authors showed a significant correlation between SR and parental depression (Carless et al. 2015; *r* = 0.44; *p* < 0.001). This effect is considered strong according to Gignac and Szodorai (2016), who recommend lower thresholds given that correlations in psychology are typically modest. Parental mental illness was a significant predictor of SR (Marin et al. 2019; *B* = 0.06; *p* = 0.01). This unstandardised coefficient effect is considered weak. The comparison between unstandardised beta coefficients and correlation coefficients reported above is limited, as unstandardised beta coefficients are scale‐dependent (depending on the unit of measurement of the predictor variable), whereas correlation coefficients are scale‐independent. Therefore, only the interpretation of the coefficients' strengths can be compared. The absence of longitudinal studies is noticed, as well as the heterogeneity of designs between studies. The limited number of robust studies including a clinical and a control group is noticed.

These results are corroborated by literature reviews, which report significant correlations between SR and parental illness (Leduc et al. 2024), identify parental mental illness as a predictor (Tekin and Aydın 2022) or a risk factor to develop SR (Chockalingam et al. 2023; Ingul et al. 2019; Ulaş and Seçer 2024). Reflexive articles are consistent with these findings, supporting the notion that parental illness is a family characteristic frequently reported in studies of children with SR (Al Husni Al Keilani and Delvenne 2021; Fremont 2003; Havik and Ingul 2021). Several studies considered parental illness as either a risk factor to develop SR (Holzer and Halfon 2006) or a contributing or maintaining one (Nuttall and Woods 2013; Prabhuswamy 2018).

Among the 18 studies included in this review, seven failed to propose potential explanatory factors for this link (Al Husni Al Keilani and Delvenne 2021; Birraux 2014; Fremont 2003; Havik and Ingul 2021; Holzer and Halfon 2006; Özcan et al. 2006; Tekin and Aydın 2022). Two others formulate an imprecise hypothesis: Children are affected by or concerned about the parental illness (Nuttall and Woods 2013; Ulaş and Seçer 2024). Several studies suggest a genetic, biological and conditioning transmission of the parental illness to the child (Bahali et al. 2011; Chockalingam et al. 2023; Ingul et al. 2019; Marin et al. 2019), or alternatively the mediation of parental emotion dysregulation between parental illness and SR (Chen et al. 2024; Chockalingam et al. 2023; Hughes et al. 2022). When parental emotional dysregulation was taken into account, parental anxiety and depression were no longer a significant explanatory factor (Chen et al. 2024). However, a second study tested this hypothesis and found no significant result (Hughes et al. 2022). A related hypothesis is that ill parents may cope in maladaptive ways with their child's SR (Bahali et al. 2011; Carless et al. 2015; Chockalingam et al. 2023; Ingul et al. 2019; Leduc et al. 2024; Marin et al. 2019). All of these hypotheses explain the link between parental mental illness and SR but not between physical illness and SR. The only hypothesis that could explain both physical and mental illness is having a carer's role (Prabhuswamy 2018).

The link between SR and parental illness is mostly considered to be unidirectional, from parental illness to child or adolescent SR (Bahali et al. 2011; Chen et al. 2024; Havik and Ingul 2021; Hughes et al. 2022; Marin et al. 2019; Prabhuswamy 2018). Nevertheless, all quantitative studies included in this review are cross‐sectional and cannot establish any directionality. Some authors suggest a potential bidirectional link might exist without testing it (Carless et al. 2015; Chockalingam et al. 2023).

## 4 Discussion

Although few studies focus on parental illness in the SR literature, this narrative review shows that this link exists and it may be strong when studied. Among the 18 articles included here, most (11 out of 18) merely acknowledged parental illness and only two studies considered it as the main topic. This association is observed in both quantitative and qualitative studies and seems to correspond to clinical reality. Nevertheless, few robust studies exist and provide precise information on the type of parental illness, the gender of the ill parent or the manner in which the illness has been diagnosed in the SR literature. Moreover, the absence of longitudinal studies and the heterogeneity of designs between studies are noted.

A link between parental mental illness and SR is more frequently observed than between parental physical illness and SR. Parental mental illness is characterised as depression and/or anxiety or not characterised at all. No information is given about other possible mental illnesses. No study focuses specifically on physical illness: studies either focus on mental and physical illness (Bahali et al. 2011; Leduc et al. 2024; Prabhuswamy 2018) or are unspecific (Holzer and Halfon 2006; Nuttall and Woods 2013), providing no information on the type of physical illness. While the link between SR and physical illness appears to exist, with 89% of mothers and 88% of fathers of SR children having a history of physical illness compared with 11% and 13% in non‐SR children (Bahali et al. 2011), this link is particularly understudied. Only three studies out of 18 focus on the gender of the ill parent. When gender is provided, mothers appear to be slightly more likely to be ill than fathers. The manner in which parental illness is diagnosed is often unspecified; the inclusion criteria were based on the authors' qualification of the illness as exceeding the pathological threshold; yet, information on how the diagnosis was established remains lacking.

Few studies provide explanations of the relationship between SR and parental illness. Seven studies do not formulate any hypothesis on this link and two others formulate imprecise hypotheses. The link between parental illness and SR is often considered from the parent to the child. Hypotheses on genetic, biological and conditioning transmission of the parental illness to the child (Bahali et al. 2011; Chockalingam et al. 2023; Ingul et al. 2019; Marin et al. 2019) are put forward. In fact, a genetic predisposition to anxiety disorders including SR is found in the literature (Hettema et al. 2001; Merikangas et al. 1999; Nursalim et al. 2018). Parental emotion dysregulation (Chen et al. 2024; Chockalingam et al. 2023; Hughes et al. 2022) or parental use of dysfunctional coping strategies with their child's SR (Bahali et al. 2011; Carless et al. 2015; Chockalingam et al. 2023; Ingul et al. 2019; Leduc et al. 2024; Marin et al. 2019) might explain the transmission of parental mental illness to the child. In fact, these parents may use more avoidant strategies to cope with SR than parents without emotion dysregulation (Carless et al. 2015). In one of the earliest definitions of SR, characterised as school phobia, Bowlby (1973) described children and adolescents as ‘those who fear the absence or loss of the person they are attached to, or being away from the environment in which they feel safe, rather than the fear and avoidance of the actual school situation’ (Eroglu et al. 2024, 9). The fear of loss is closely linked with the experience of having an ill parent. Some study cases of children and adolescents with SR mention the concept of separation anxiety (Catheline 2019; Hanna et al. 2016; Holzer et al. 2018). Separation anxiety is defined as the fact of ‘[fearing] something bad will happen to them or someone they love (usually parent or caregiver) when they are apart’ (Bagnell 2011, 442). Separation anxiety is particularly activated in children or adolescents in the situation of parental mental illness (Hanna et al. 2016). Nevertheless, it is important not to reduce SR to separation anxiety (Harf et al. 2022). Separation anxiety is mostly considered as a predictor of SR or as a comorbidity (Nair et al. 2013; Tekin et al. 2018). It might also represent a maintaining factor of SR (Holzer et al. 2018) by staying close to the parent when not going to school or by obtaining their attention (Tekin and Aydın 2022).

Moreover, an alternative explanatory hypothesis of this link is the caregiving situation (Prabhuswamy 2018). In fact, having a parent who is ill can lead to becoming a carer and more precisely a young carer (YC; Dearden and Becker 2004). YC are children and adolescents who provide meaningful, regular support to a relative with a health problem (American Association for Caregiving Youth 2012). The number of YC ranges from 7.9% to 14.3% depending on age and country (Leu et al. 2018; Untas et al. 2022). This situation has several repercussions on their developmental trajectory and in particular on their mental health and school life (Becker and Sempik 2019). YC are more likely to have difficulties at school (Dearden and Becker 2004), poorer mental health (Lacey et al. 2022) and particularly more anxiety disorders (Jarrige et al. 2020) compared with noncarers. The link between absenteeism and caregiving situation has already been observed: caregivers' responsibilities are considered as risk factors of chronic absenteeism (Allen et al. 2018). Nevertheless, the caregiving situation has never been considered as a risk factor for SR and deserves further investigation, alongside other potential explanations. Indeed, no study included in this review directly tested the YC hypothesis or its relationship with SR. The supporting evidence for this hypothesis is mentioned in the literature (Prabhuswamy 2018) and is corroborated by findings from adjacent literatures, such as studies on absenteeism and general anxiety among YC (Dearden and Becker 2004; Jarrige et al. 2020). As such, it should be considered as a theoretical perspective rather than a definitive finding of this review. To empirically test this hypothesis, future studies should compare SR scores between YC and non‐YC adolescents.

This narrative review has clinical implications. Including parents in SR therapy has been proven to be more efficient than individual therapy (Carr 2019; Chockalingam et al. 2023). This is even truer when the parent has a mental illness (Carr 2019) as parental mental illness seems to influence SR therapy negatively. Both parental mental illness (Bahali et al. 2011) and child SR must be treated conjointly, as these conditions may influence each other bidirectionally (Carless et al. 2015; Chockalingam et al. 2023). Moreover, when the child or adolescent is a YC, their carer role and its specificities must be considered in treatment. In fact, YC may have specific needs (Aldridge 2008), which must be taken into account when supporting them. Nowadays YC are rarely identified and their needs are rarely met (Blake‐Holmes and Cook 2024; Leu and Becker 2017).

This narrative review has several limitations. First of all, this narrative review did not follow systematic review guidelines, which might represent a methodological limitation. Moreover, several systematic reviews and literature reviews were included in this review (Chockalingam et al. 2023; Ingul et al. 2019; Leduc et al. 2024; Tekin and Aydın 2022; Ulaş and Seçer 2024). Several primary studies are shared across these reviews, resulting in potential double‐counting of findings. Therefore, the conclusions drawn from this review are ultimately based on a limited pool of original studies, reflecting the fact that this topic has not yet been explored in depth. Secondly, SR diagnosis relies mostly on clinical teams without specification of the decision‐making criteria, which may constitute a bias. Future research using standardised psychometric scales to assess SR in nonclinical populations is needed in order to study the relationship between parental illnesses and SR in a dimensional perspective among children and adolescents prior to clinical treatment. Most importantly, the interpretation of the relationship between parental illnesses and SR is often suggested to be from the parent to the child but this might be a methodological artefact and directionality of the link should be investigated with longitudinal data in the future. Moreover, most studies included in this review are quantitative. However, qualitative studies using a narrative approach could be interesting in order to shed light on the place of parental illness in SR.

## 5 Conclusion

The present narrative review has underlined that while parental illness and its link with SR are mentioned in the literature, it is rarely the main focus of study. This also highlights the lack of precise data on parental illnesses in the SR literature and the limited explanatory hypotheses proposed to explain this link, especially regarding parental physical illness. Studying this link is crucial in order to support these children or adolescents and their families effectively. The hypothesis of the caregiving situation as a risk factor of SR requires greater attention. Treatment of SR should address not only parental illness, if relevant, but also the caregiving situation.

## Author Contributions

**Bruno Dauvier:** conceptualization, writing – review and editing, visualization, validation, methodology, software, formal analysis, project administration, supervision, resources, funding acquisition. **Basilie Chevrier:** resources, supervision, project administration, formal analysis, software, methodology, validation, visualization, writing – review and editing, conceptualization, funding acquisition. **Salomé Villetorte:** conceptualization, investigation, funding acquisition, writing – original draft, writing – review and editing, methodology, validation, visualization, software, formal analysis, project administration, data curation.

## Funding

This work was supported by Aix‐Marseille Université with a grant for a PhD.

## Conflicts of Interest

The authors declare no conflicts of interest.

## Data Availability

Data sharing is not applicable to this article as no datasets were generated or analysed during the current study.
